# Somatostatin receptors in pituitary somatotroph adenomas as predictors of response to somatostatin receptor ligands: A pathologist's perspective

**DOI:** 10.1111/bpa.13313

**Published:** 2024-10-30

**Authors:** Laura Botelho, Rômulo Sperduto Dezonne, Luiz Eduardo Wildemberg, Renan Lyra Miranda, Mônica R. Gadelha, Felipe Andreiuolo

**Affiliations:** ^1^ Neuropathology and Molecular Genetics Laboratory Instituto Estadual do Cérebro Paulo Niemeyer Rio de Janeiro Brazil; ^2^ Department of Pathology Rede D'Or Rio de Janeiro Brazil; ^3^ Neuroendocrinology Research Center, Endocrinology Division Medical School and Hospital Universitário Clementino Fraga Filho, Universidade Federal do Rio de Janeiro Rio de Janeiro Brazil; ^4^ Neuroendocrinology Division Instituto Estadual do Cérebro Paulo Niemeyer Rio de Janeiro Brazil; ^5^ D'Or Institute for Research and Education Rio de Janeiro Brazil

**Keywords:** immunoreactivity score, pituitary adenoma, somatostatin receptor ligands, somatostatin receptors, somatotropinomas, SST2

## Abstract

There are five subtypes of somatostatin receptors (SST1‐5), which are expressed in several types of solid neoplasms, neuroendocrine tumors, and pituitary adenomas. Most commonly, SST2 and SST5, are of interest regarding diagnostic, treatment, and prognostic purposes. In this article the basic biological characteristics of SST are briefly reviewed, and focus given to the immunohistochemical evaluation of SST2 and SST5 in growth hormone (GH)‐secreting pituitary tumors, and their quantification as predictors of response to treatment with somatostatin receptor ligands (SRL), the mainstay of the pharmacological therapy available for these tumors. Although many different scoring systems for SST2 immunohistochemistry showing correlation with SRL response have been reported, among which the immunoreactivity score (IRS) has been the most consistently used, a universally validated immunohistochemical technique and scoring scheme is lacking. Efforts should be made on collaborative multicenter studies aiming at validating homogeneous immunostaining protocols and a scoring system for SST2 and SST5 expression, to help clinicians to define the optimal therapeutic strategy for the patients with somatotroph tumors.

## Graphical abstract

This article reviews the major biological characteristics of somatostatin receptors (SST2 and SST5) in anterior pituitary tumors, and the immunohistochemical evaluation of their expression as predictors of response to pharmacological therapy with SST ligands.

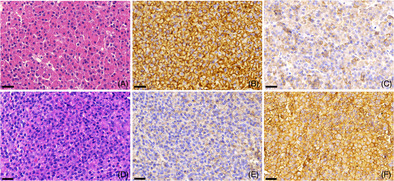

## 1 INTRODUCTION

Somatostatin is a polypeptide hormone with a broad range of actions, including the inhibition of hormone release, cell growth, and differentiation and exerts its intracellular effects by binding to somatostatin G protein‐coupled receptors (SST) [1]. There are five SST subtypes (SST1‐5), which are expressed in different types of solid neoplasms and several neuroendocrine tumors (NETs) [1], the most common subtypes being SST2 and SST5 [2]. In diagnostic pathology, SST2 has been widely used as a sensitive immunohistochemical marker for meningiomas and is frequently expressed in olfactory neuroblastomas [3, 4, 5, 6]. Besides the interest as a diagnostic marker, the expression of SST2 has also shown prognostic value in several types of tumors, for instance being associated with better outcome in pancreatic and rectal NETs and nasopharyngeal carcinoma [5, 6, 7, 8]. In NETs, the expression of SST2 by tumor cells is also of interest for the therapeutic strategy, as it can be used as a target for radiolabeled imaging and for targeted therapy with labeled analogs [9, 10].

The most recent World Health Organization classification of Tumors of Endocrine Organs (2022) encourages the use of the term Pituitary Neuroendocrine Tumor (PitNET) to denominate tumors of adenohypophysial hormone‐secreting cells, nonetheless, the term adenoma still can be used. According to our practice we have chosen to keep with the latter throughout this manuscript [11]. The main therapy for pituitary adenomas is surgery, except for prolactin secreting adenomas, however, even in specialized centers, a significant proportion of patients will need adjuvant therapy [12, 13]. For patients with functioning adenomas after unsuccessful surgery (or for those for whom surgery was not an option), medical management is the cornerstone of treatment [13]. In this setting, somatostatin receptor ligands (SRL), which exert their action by interacting with SST, are an important class of drugs for the treatment of adenomas secreting growth hormone (GH), adrenocorticotropic hormone (ACTH), and thyroid‐stimulating hormone (TSH) [13]. It has been demonstrated that the efficacy of these drugs correlates with the expression of SST in the adenomas, especially in acromegaly [14, 15, 16].

Therefore, the immunohistochemical evaluation of SST2 and SST5 expression in pituitary adenomas may be useful to determine the most appropriate drug for each patient, improving response rates, and pathologists have been requested to perform this task. Although different scoring systems have been successfully employed [16, 17, 18, 19, 20], there is to this date no standardization on a specific technique for immunostains and no consensus on a scoring system which should be used.

This article reviews briefly the basic biological characteristics of SST and selected studies focusing on their immunohistochemical evaluation in pituitary adenomas, and their quantification as predictors of response to treatment with SRL [15].

## 2 BIOLOGICAL CHARACTERISTICS OF SOMATOSTATIN RECEPTORS

Somatostatin is a hormone that acts as an inhibitory regulator of hormones' secretion and cell proliferation. As previously mentioned, this action is mediated by a family of G protein‐coupled transmembrane domain receptors, which consist of five distinct subtypes SST1‐5 [21]. Natural forms of somatostatin, somatotropin‐release inhibitory factors (SRIF or somatostatins), SRIF14 and SRIF28 bind to all receptors with high affinity. However, except for SST5, which has higher affinity for SRIF28, the other receptors have higher affinity for SRIF14 [22]. Furthermore, the SSTs isoforms have an affinity with corticostatin, a peptide with high similarity to somatostatin [21]. There are several SRL, each one with different binding affinities to SST. The clinically available ones are the first‐generation SRL (fg‐SRL), octreotide and lanreotide, which bind with high affinity to SST2 and to a lesser extent to SST5, and pasireotide, which binds with higher affinity to SST5, followed by SST2, SST3, and SST1 [15].

The SST are G protein‐coupled receptors that have seven transmembrane domains (TMDs) [23]. All SST are expressed in the anterior pituitary during development, in different degrees. Of note, SST2 is also expressed in different regions of the human cerebrum, cerebellum, neuroendocrine cells of the gastrointestinal system, endocrine pancreas, reticular zone of adrenal cortex, kidney, ovary, testis, parotid and endothelial cells, and other tissues [24, 25]. SST5 is also present in these tissues, but with much more limited expression in the central nervous system [24, 25].

**FIGURE 1:**
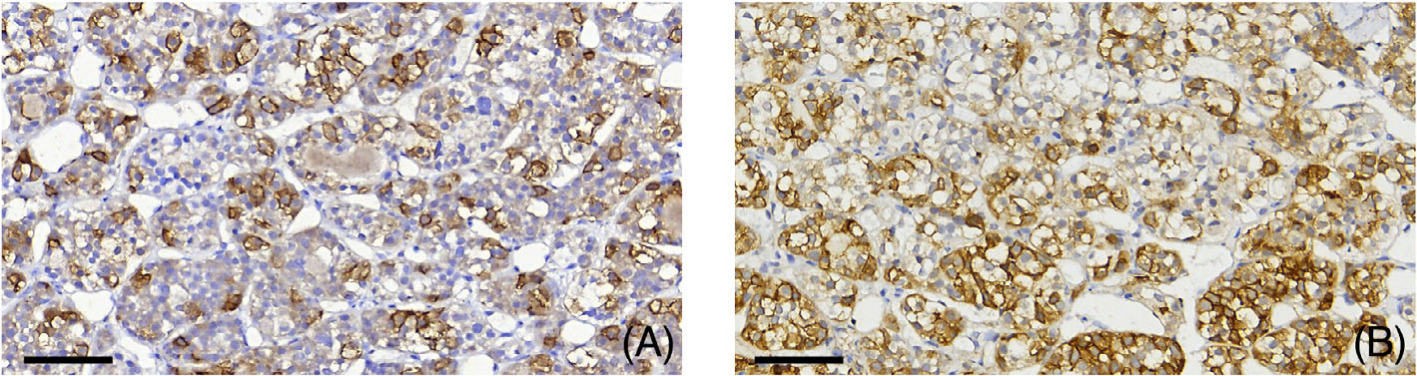
SST2 and SST5 IHC in non‐neoplastic anterior pituitary. SST2 (A) and SST5 (B) immunostains showing heterogeneous positivity. Scale bars measure 60 μm.

In the adult adenohypophysis, the isoforms expressed are SST1, 2, 3, and 5, with SST2 and SST5 being the most representative [26] (Figure 1). SST2 is expressed mostly by somatotrophs and to a lesser extent by gondadotrophs, corticotrophs, thyrotrophs, and lactotrophs [24]. The SST2 is encoded by the *SSTR2* gene that has two exons and can generate two isoforms, SST2A and SST2B with 369 and 346 amino acids, respectively. SST2A is the most widely expressed isoform [27]. The *SSTR5* gene has two exons and normally produces two transcript variants that are translated to the same 364 amino acid full‐length protein. In addition, there are two truncated isoforms of the SST5 produced by alternative splicing, one with five TMDs known as the sst5TMD5 and the other with four TMDs known as sst5TMD4 [28]. In contrast to the full‐length receptor, the shorter isoforms (273 and 216 amino acids) are predominantly intracellularly located [28]. The isoform sst5TMD5 is expressed in normal tissues, and sst5TMD4 is expressed in very low levels. These truncated isoforms respond differently to somatostatin and corticostatin [28].

Previously, sequencing of SST2 and SST5 did not reveal pathogenic variants in the coding and bordering regions in somatotroph tumors resistant to somatostatin ligands [29]. The variant R240W of the SST5 was described in a patient with acromegaly who was resistant to fg‐SRL, and in vitro analysis showed that cells carrying the variant had a reduced response to octreotide [30]. Also, the presence of sst5TMD4 was demonstrated in patients that expressed SST2 but were resistant to octreotide and responded to pasireotide [31].

### 2.1 Somatostatin receptor signaling pathways

A classic effect of SST on the somatotrophs is the inhibition of GH secretion. In this context, SST2 and SST5 play a significant role. However, other studies have already demonstrated the inhibition of both ACTH and TSH secretion by SST [32]. In addition to the classic antisecretory effect, the activation of SSTs in the anterior pituitary regulates other relevant physiological effects such as apoptosis and proliferation [26].

#### 2.1.1 Antisecretory pathways

**FIGURE 2:**
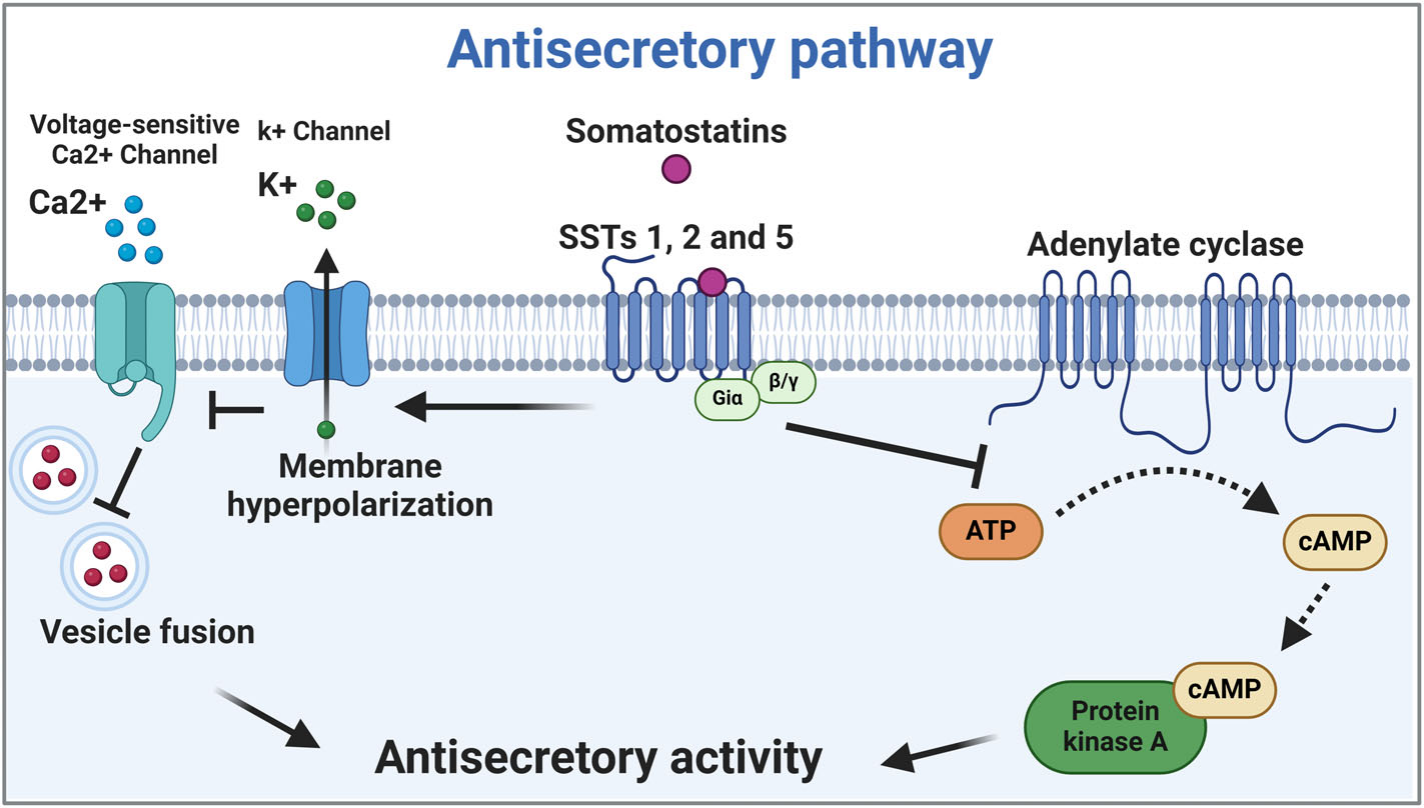
SSTs' antisecretory pathways. The major antisecretory SSTs pathway involves adenylate cyclase inhibition, consequently, cyclic AMP (cAMP) decrease and protein kinase A (PKA) suppression. SSTs also activate various types of potassium channels, allowing K+ efflux and plasma membrane hyperpolarization. Membrane hyperpolarization blocks the depolarization‐induced Ca^2+^ influx via voltage‐sensitive Ca^2+^ channels and vesicle fusion at plasma membrane. All these pathways will result in the inhibition of hormone secretion. ATP: adenosine triphosphate; cAMP: cyclic adenosine monophosphate; SSTs: somatostatin receptors. Created with BioRender.com.

SST are coupled to inhibitory G proteins (Giα) that suppress the activity of the enzyme adenylate cyclase (AC) and, consequently, the levels of cyclic AMP (cAMP), the activator of the cyclic AMP‐dependent protein kinase enzyme A (PKA) [33] (Figure 2). In this context, the main antisecretory pathway of SST is dependent on AC inhibition [34, 35]. SST1, SST2, and SST5 inhibit GH secretion [36, 37]. However, the antisecretory function is not exclusively dependent on PKA inhibition, as SSTs also activate various types of potassium (K+) channels, allowing K+ efflux, resulting in hyperpolarization of the plasma membrane and, consequent block of the depolarization‐induced Ca2+ influx via voltage‐sensitive Ca2+ channels (Figure 2). As secretory vesicles fusion at plasma membrane is dependent on calcium, the activation of K+ channels would result in the inhibition of GH secretion [38, 39]. In this scenario, the only isoform that is not coupled to potassium channels is SST3 [38].

#### 2.1.2 Antiproliferative pathways

**FIGURE 3:**
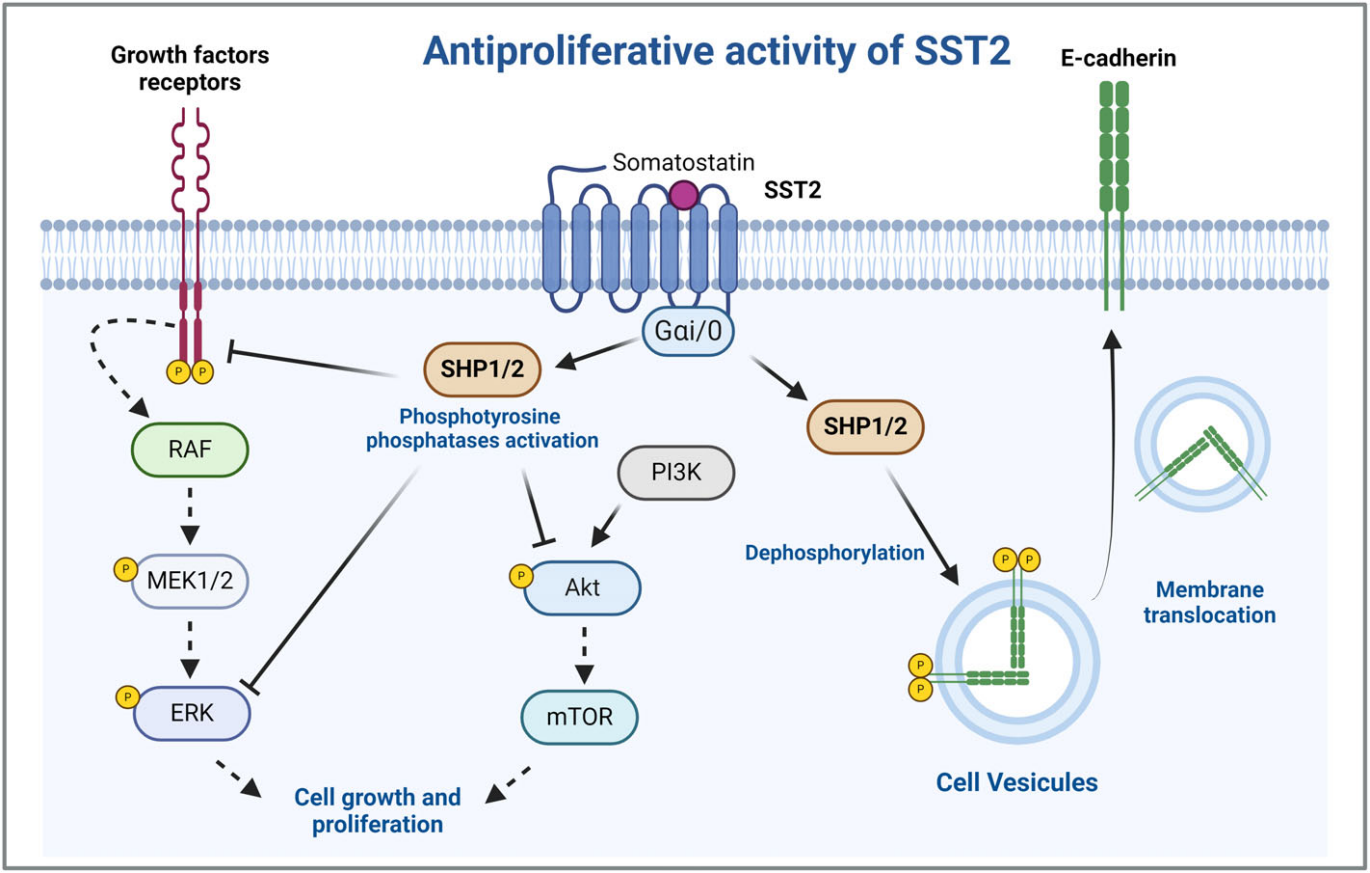
SSTs' antiproliferative activity. SSTs inactivate growth factors’ tyrosine kinase receptors and its downstream effector ERK, by activation of phosphotyrosine phosphatases SHP‐1 and SHP‐2. In addition, SST2 inhibits PI3K pathway at Akt. SST2 by SHP1‐ and SHP‐2 phosphatases also removes the phosphates from E‐Cadherin’s intracellular domain, which allows its translocation to plasma membrane. Akt: Protein kinase B, PKB; ATP: adenosine triphosphate; BCL‐2: B‐cell leukemia/lymphoma 2 protein; cAMP: cyclic adenosine monophosphate; ERK: Extracellular signal‐regulated kinase; MEK: Mitogen‐activated protein kinase kinase; mTOR: Mechanistic target of rapamycin; PI3K: phosphoinositide 3‐kinase; RAF: rapidly accelerated fibrosarcoma kinase; SHP‐1/2: phostyrosine phosphatases SHP‐1 and SHP‐2; SST2: somatostatin receptors 2. Created with BioRender.com.

Regarding the antitumor effects of SST, they have a crucial role in the inactivation of tyrosine kinase receptors, activated by growth factors and essential in several signaling processes. In the presence of its ligands, SSTs activate phosphotyrosine phosphatases (PTP), SHP‐1 and SHP‐2 [40], that inactivate the intracellular portion of these receptors, blocking their activation cascade and arresting the cell at G1, especially SST1, SST2, and SST5 [41, 42]. These effects are mediated by activation of two different G proteins, Gαi and G0 [43]. Furthermore, SST2 also inhibits phosphatidylinositol 3‐kinases (PI3K), by activation of SHP‐1 and SHP‐2, an important pathway involved in cell survival and growth (Figure 3). This suppression occurs at serine/threonine‐specific protein kinase Akt [1]. SST2 also dephosphorylates E‐cadherin, restoring its functions in plasma membrane (Figure 3).

**FIGURE 4:**
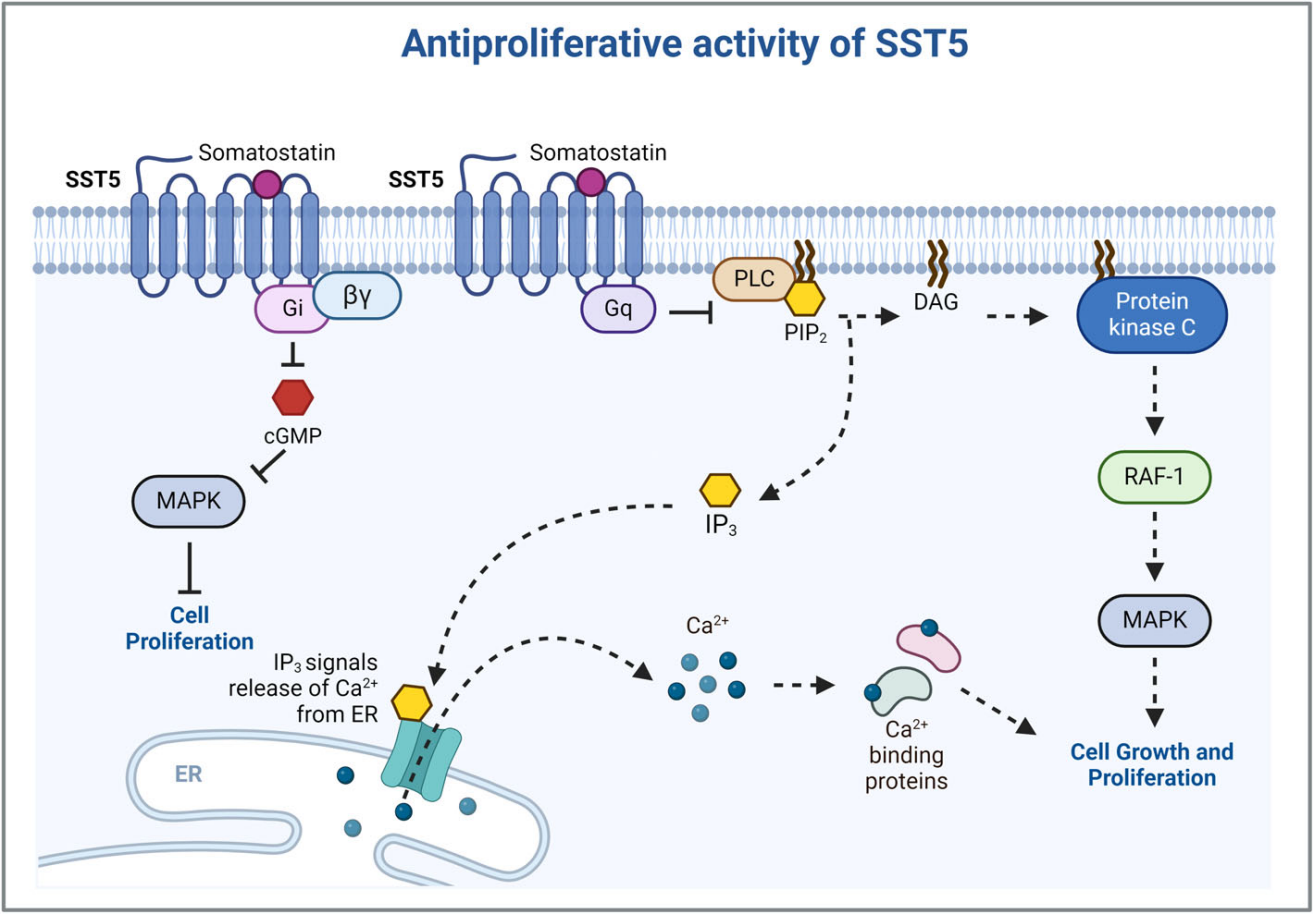
SST5's antiproliferative pathways. SST5 inhibits Phospholipase C (PLC), inositol 1,4,5‐trisphosphate (IP3) and diacylglycerol (DAG) and, consequently, blocks Ca^2+^ dependent pathways. In addition, by decreasing cGMP levels, SST5 activation also leads to inhibition of the MAPK pathway and proliferation. cGMP: cyclic guanosine monophosphate; MAPK: mitogen‐activated protein kinase; RAF‐1: Raf‐1 proto‐oncogene, serine/threonine kinase; SST5: somatostatin receptor 5. Created with BioRender.com.

Compared to SST2, SST5 activation uses alternative non‐PTP‐dependent pathways. One of the main pathways triggered by SST5 activation is the inhibition of phospholipase C (PLC), inositol 1,4,5‐trisphosphate (IP3), and diacylglycerol with the consequent blockage of Ca2+ influx [44, 45, 46]. Furthermore, activation of SST5 also leads to inhibition of the MAPK pathway and proliferation (Figure 4) [47].

#### 2.1.3 Pro‐apoptotic pathways

**FIGURE 5:**
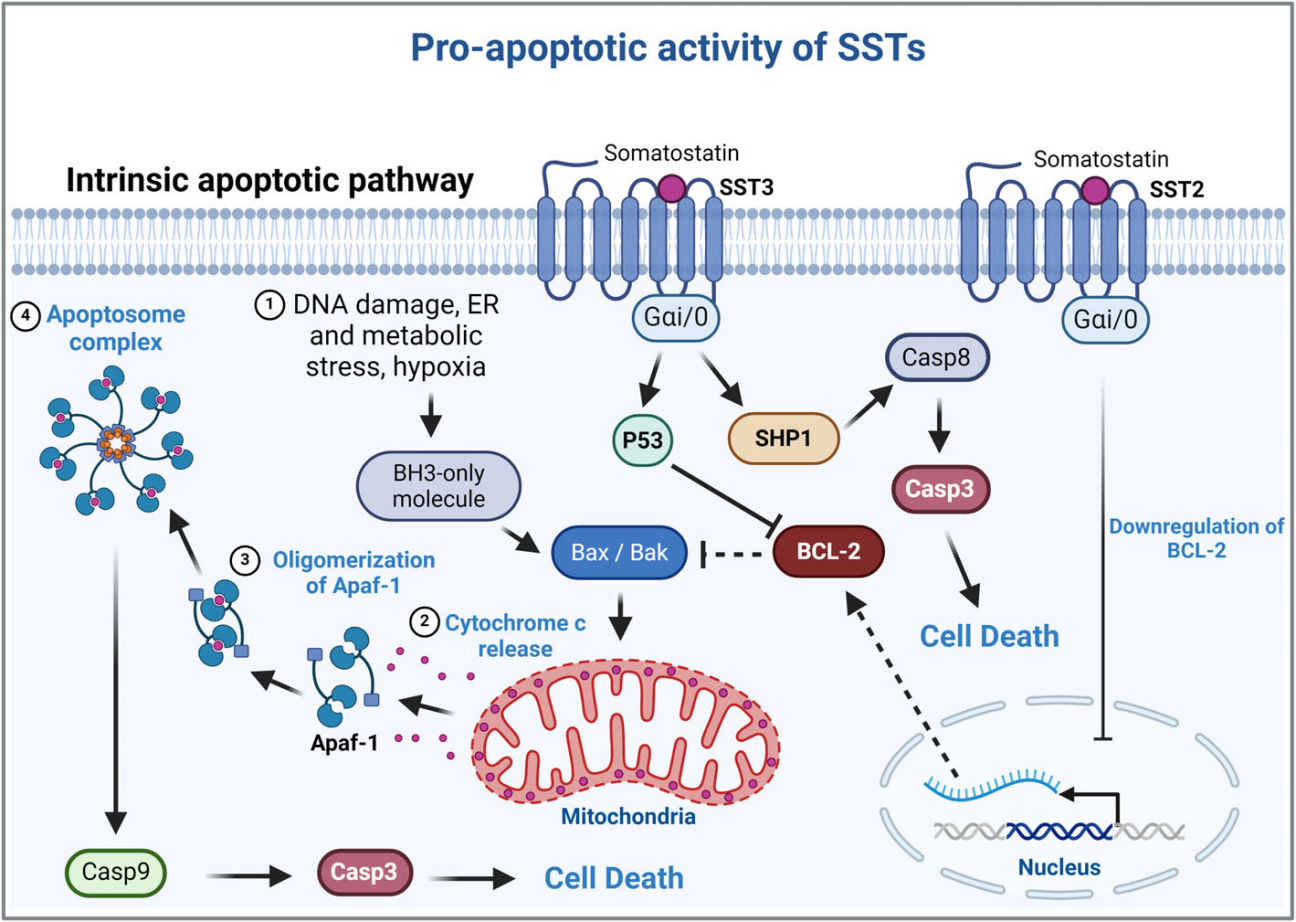
SSTs' pro‐apoptotic pathways. 1) The intrinsic pathway is activated by DNA damage and other forms of cellular stress, which result in BH3‐only activation and Bax/Bak oligomerization and pore formation in the mitochondrial outer membrane. 2) The mitochondrial membrane permeabilization promotes release of cytochrome c. 3) Cytosolic cytochrome c accumulation promotes protein apoptotic protease activating factor 1 (Apaf‐1) oligomerization and 4) Apoptosome complex formation. The Apoptosome acts on procaspase‐9, allowing its conversion into the active form Caspase‐9 (Casp9), which activates the executioner Caspase‐3 (Casp3) and triggers cell death. SST3 induces cell death via activation of p53 protein, and consequent inhibition of BCL‐2. In addition, SST3, mediated by phosphotyrosine phosphatase SHP‐1, induces the activation of Casp3 via Caspase‐8 (Casp8). On the other hand, SST2 suppresses BCL‐2 transcription, decreasing its inhibition on Bax. Apaf‐1: protein apoptotic protease activating factor 1; Bak: Bcl‐2 homologues antagonist/killer; Bax: Bcl‐2–associated X protein; SHP‐1: phosphotyrosine phosphatase protein SHP‐1. Created with BioRender.com.

Apoptosis can be induced by the intrinsic and the extrinsic pathways that lead to activation of caspases [48]. One of the classical apoptosis pathways, the intrinsic pathway, requires mitochondrial membrane permeabilization (MMP). The MMP occurs after conformational changes in the proteins BAX and BAK that result in their oligomerization and pore formation in the outer mitochondrial membrane, which promotes cytochrome c release [49] and activation of caspase‐9, which triggers the executioner caspase‐3 to induce apoptosis [48]. Alternatively, caspase‐3 can be activated by the initiator caspase‐8 [50]. In this context, SST3 can stimulate apoptosis, mediated by the activation of p53 and BAX. In addition, recruitment of SHP‐1 leads to activation of Caspase 8 [51]. SST2 also downregulates BCL‐2 [51], which suppresses pro‐apoptotic proteins BAX and BAK (Figure 5).

## 3 SST DETECTION METHODS

Initial studies on SSTs evaluated their messenger RNA (mRNA) expression pattern using RT‐PCR findings [52]. However, analysis by this method does not evaluate the functional expression of the protein in the cell membrane as it does not discriminate subcellular localization [52]. Several other methods have been used to analyze SST expression in NET, including in situ hybridization, scintigraphy, in vitro autoradiography, immunoblotting, and IHC [2, 53, 54, 55, 56]. IHC was first tested in neoplasms by Reubi et al., who compared this method of detection with autoradiography and in situ hybridization, validating its efficacy for the analysis of SST2 expression in tumors (including somatotroph and nonfunctioning pituitary adenomas) [54]. IHC offered advantages over the other methods, such as the visualization of the actual labeling in the neoplastic cell, the exclusion of labeling in endothelial cells and a more accurate scoring, in addition to greater practicality and shorter execution time [54]. Further studies validated the immunohistochemical analysis of SST2 in comparison with RT‐qPCR, which demonstrated a positive correlation of the immunohistochemical expression of SST2 with the expression of SST2 mRNA [57, 58, 59].

### 3.1 Immunohistochemistry

The main antibodies against SST2, SST3, and SST5 reported in the literature are depicted in Table S1.

Volante et al. analyzed the expression of three different polyclonal SST antibodies in 106 NET and one single pituitary adenoma and observed a greater sensitivity of the SS‐800 antibody both in labeling the internal control (blood vessels) and the cell membrane of normal and neoplastic cells. The first studies on somatostatin receptors in series of anterior pituitary tumors used polyclonal antibodies from different manufacturers, all with manual immunohistochemical techniques [17, 19, 59, 60, 61]. The monoclonal antibody clone UMB‐1 binds to the carboxy‐terminal portion of SST2, located in the inner (cytoplasmic) leaflet of the plasma membrane and UMB‐4 antibody binds to the carboxy‐terminal portion of SST5 [24]. UMB‐1 was tested in 2008 in NET (the study also included two somatotroph adenomas) and was considered superior to polyclonal antibodies, because of the prominent membrane staining and low cytoplasmic staining [62]. The first study specifically dedicated to the scoring of SST2 in pituitary adenomas using monoclonal antibodies was published in 2013 by Gatto et al., and since then, monoclonal antibodies have been consistently utilized, mostly with automated techniques (Table S2) [18, 62, 63, 64, 65, 66, 67, 68, 69, 70]. Although all primary monoclonal antibodies in the studies here reviewed are from the UMB1 clone, the dilution used was highly variable (from 1:25 to 1:5000). However, if one takes into consideration antibodies from the same manufacturer, this variation is less striking, (Biotrend, 1:25 to 1:50), (Epitomics 1:100), (Clinisciences 1:4000), (Abcam 1:500 to 1:5000) (Table S2), and probably reflects individual products' properties.

Chinezu et al. have used both manual (avidin–biotin based—Histostain‐Plus Bulk kit; Invitrogen, Camarillo, CA) and automated (Benchmark XT, Ventana Medical Systems) immunohistochemical techniques with SST2A and SST5 monoclonal antibodies (UMB‐1 and UMB‐4, respectively, Abcam), which showed no significant difference in the SST2 expression in somatotroph adenomas preserved with Bouin's fixative, but for the SST5 antibody, the intensity and the percentage of immunoreactive neoplastic cells was lower with the manual technique [62]. Indeed, it is well documented that several factors such as fixative agent and fixation time, tissue processing, antigen retrieval methods, and environmental conditions can significantly impact the performance of IHC. Therefore, the College of American Pathologists advocates specifically for the semiquantitative/numerical scoring of predictive markers, that a minimum of 20 positive and 20 negative tissues are tested, spanning the whole range of expected clinical results [71]. Although somatotroph adenomas correspond to around 10%–15% anterior pituitary surgical specimens [11], pathology laboratories outside Reference Centers in Neuroendocrinology will most likely not have enough samples with clinical data available for correlation, to be able to ensure an adequate assessment of SST2 IHC [72]. For instance, in our Institution (IEC PN), where in the last 10 years more than 1000 pituitary surgeries were performed, only around 20–30 patients with acromegaly are operated on each year.

#### 3.1.1 Immunohistochemistry scoring systems

Many studies have applied immunohistochemical analysis of SST in pituitary adenomas, which correlated with the treatment response with SRL. Concise comparative data are summarized in Table 1 and more detail is depicted in Tables S2 and S3. We have selected studies on pituitary adenomas which, through different SST2 immunohistchemical scoring systems, obtained relevant/interesting results. We found a total of 10 different scoring systems, as described below.

**TABLE 1:** Main scoring systems for SST2 immunohistochemical assessment in the literature.

Author	Scoring system	Staining pattern assessed
Thodou 2006 [20]	HER‐2 score (membrane staining): 0 (negative or <10% positive); 1+ (faint or incomplete in more than 10%); 2+ (weak to moderate in more than 10%); and 3+ (strong and complete in more than 10%)	Membrane
Volante 2007 [60]	0: absence of staining; score 1: exclusively cytoplasmic staining (focal or diffuse); 2: (membrane staining in <50% tumor cells, independently from cytoplasmic staining); and 3: circumferential membrane staining in more than 50% tumor cells independently from cytoplasmic staining	Membrane and cytoplasm
Takei 2007 [17]	Percentage of positive cells: 0 (negative); 1+ (<25%); 2+ (20%–50%); and 3+ (more than 50%)	Membrane
Fougner 2008 [19]	1 (<25% positive cells (all intensities)); 2 (25%–75% positive, any intensity or >75% of cells stained with weak intracytoplasmic positivity); and 3 (>75% of cells stained with moderate to strong intensity)	Membrane and cytoplasm
Casarini 2009 [59]	Strong staining (+++), moderate staining (++), weak staining (+), and pale staining (±)	Membrane
Wildemberg 2013 [61]	Percentage of stained cells 0 (<25%); 1 (25%–50%); and 2 (>50%)	Membrane and cytoplasm
Gatto 2013 [16]	Scores from 0 to 12. IRS: product of the percentage of positive cells (4 >80%; 3, 51%–80%; 2, 10%–50%; 1, <10%; and 0, 0%) and the intensity of the staining (3, strong; 2, moderate; 1, weak; and 0, negative)	Membrane
Casar‐Borota 2013 [67]	IRS (see above)	Membrane
Chinezu 2014 [62]	Same score as Fougner, but considering only the % of immunoreactive cells and divides into three: 1: <25%; 2: 25%–75%; and 3: >75%	Membrane
Iacovazzo 2016 [70]	Volante score (see above)	Membrane and cytoplasm
Muhammad 2019 [63]	IRS (see above)	Membrane
Soukup 2021 [18]	(H‐score) Intensity from 1 to 3 multiplied by the percentage of stained cells	Membrane
Wildemberg 2021 [65]	IRS (see above)	Membrane
Rass 2022 [69]	Score 0: negativity; Score 1: Up to 20% of cells positive (+/low), Score 2: 21–50% of cells positive (++/moderate); and Score 3: more than 50% of cells positive (++ +/high)	Membrane
Ilie 2022 [68]	IRS (see above)	Membrane
Campana 2022 [66]	Digital imaging analysis. Score in continuous variable, considering number of cells, number of stained cells, intensity of the staining (total intensity: sum of all pixel intensity values), and ROI area (number of analyzed pixels)	Membrane and cytoplasm

Abbreviation: IRS, immunoreactivity score.

The first one was an adaptation of the HER‐2 score widely used in breast cancer which consists of four different groups, based on membrane staining: 0 (negative or <10% positive); 1+ (faint or incomplete in more than 10%); 2+ (weak to moderate in more than 10%); and 3+ (strong and complete in more than 10%) [20].

Volante score used a semiquantitative approach, combining the extent and subcellular location of the staining, resulting in four scores: Score 0—absence of staining; Score 1—exclusively cytoplasmic staining (focal or diffuse); Score 2—membrane staining in <50% tumor cells, independently from cytoplasmic staining; and Score 3—circumferential membrane staining in more than 50% tumor cells independently from cytoplasmic staining [60].

The scoring system used by Takei et al. separates into four groups according to the proportion of positive cells with membrane positivity: negative; 1+ (<25%); 2+ (25%–50%); and 3+ (over 50%) [17].

Fougner et al. has applied a semiquantitative scoring system, divided into three groups: Grade 1 (<25% positive cells independently from intensity); Grade 2 (25%–75% positive cells any intensity or >75% stained cells with weak intracytoplasmatic staining); and Grade 3 (>75% cells with moderate to strong staining) [19].

A semiquantitative scale was used by Casarini et al. considering the intensity of membrane staining as follows: strong (+++); moderate (++); weak (+); and pale staining (±) [59].

Our group has previously used a scoring system based on the percentage of positive cells considering both membrane and cytoplasmatic staining divided in three scores: 0 (<25%); 1 (25%–50%); and 2 (more than 50%) [61].

**FIGURE 6:**
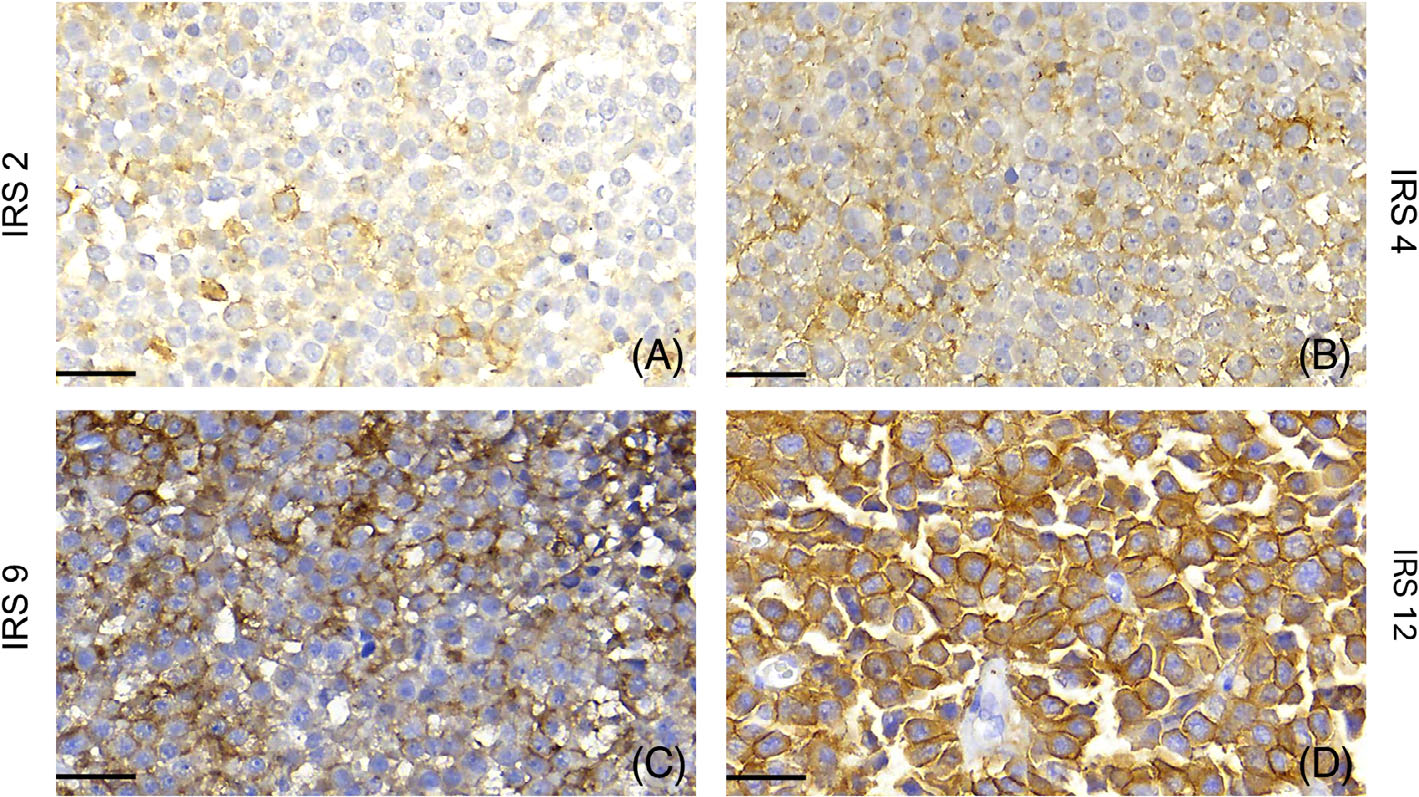
Immunoreactivity score (IRS) in somatotroph adenomas. Immunohistochemical quantification of SST2 expression. (A) IRS = 2 (2 x 1 = Moderate intensity staining in less than 10% neoplastic cells). (B) IRS = 3 (1 x 3 = Moderate staining in less than 50% neoplastic cells). (C) IRS = 9 (3 x 3 = Strong intensity staining in more than 50% neoplastic cells). (D) IRS = 12 (4 x 3 = Strong intensity staining in more than 80% neoplastic cells). All scale bars measure 30 μm.

In 2013, Gatto et al. analyzed pituitary adenomas from acromegalic patients, using the immunoreactivity score (IRS), which consists of the product of the percentage of positive cells (4 >80%; 3, 51%–80%; 2, 10%–50%; and 1, <10%; 0, 0%) and the intensity of the membrane staining (3, strong; 2, moderate; 1, weak; and 0, negative) [16]. Examples are shown in Figure 6. The IRS was initially described for evaluating the immunostaining of estrogen receptors in breast cancer (Remmele & Stegner, 1987 in Gatto) [16, 73].

Chinezu et al. adapted Fougner's scoring system but considered only the percentage of immunoreactive cells [62, 64].

The scoring system proposed by Soukup et al. (histoscore/H‐Score), is obtained by the product between the percentage of cells and their respective membrane staining intensity (a scale from 1 to 3), considering different areas in the same tumor and the sum between them, with a final score ranging from 0 to 300 [18].

Campana et al. performed digital image analysis (DIA) to quantify SST using an open‐source software (Cellprofiler), applying a continuous variable to quantify the positive tumor cells and the intensity of IHC. The DIA is preceded by two manual steps, the selection of four representative tumor areas by the pathologist and the outline of the region of interest (ROI). Then digital image analysis is performed using the software, the percentage of positive cells (%PC) is measured (calculated by dividing the number of stained cells by the total number of tumor cells) and the intensity/area calculated by dividing the sum of all the intensity and the area of the ROI. The intensity/area varied between 0 and 1 and the %PC was a continuous variable [66].

The majority of the systems included the intensity of the staining, being more specific, six included it in the analyses (HER 2‐score, the one used by Fougner et al., the one used by Casarini et al., IRS, H‐Score and DIA) [16, 18, 19, 20, 59, 67, 69] and only four (Volante score, the one used by Takei et al., the one we used in the past, and the one used by Chinezu et al.) considered only the percentage of stained cells [17, 60, 61, 62]. Regarding the incorporation of the intensity of staining, versus only the percentage of positive cells stained, Campana et al. found that the proportion of stained cells demonstrated greater accuracy in predicting response to pharmacological treatment when compared to the IRS (which also considers the intensity of the staining) [66]. Of note, the studies by Chinezu et al., Takei et al., and Wildemberg et al. considered only the percentage of stained cells positive for SST2 [17, 58, 62]. Moreover, looking closely at the studies by Casar‐Borota and Ilie where the significant IRS cutoff for treatment response prediction equaled 7 or more [67, 68], only tumors with more than 50% stained cells achieve this value. Nonetheless, the percentage of positive cells for SST2 considered for correlation in the different studies varied (50% by Takei et al., 68% by Campana et al., and 75% by Chinezu et al.) [17, 62, 66]. A previous study from our group still using polyclonal SST2 antibodies revealed that <25% tumor cells positive for SST2 predicted the lack of biochemical response to pharmacological treatment [61].

Among 18 studies, 5 considered membrane and cytoplasmatic expression [19, 60, 61, 66, 69] when evaluating the SST2 expression and 13 of them included membrane expression exclusively [16, 17, 18, 20, 62, 63, 64, 65, 67, 68, 69, 74]. Regarding the 10 scoring systems, 4 included the cytoplasmic staining (Volante score; Fougner; Wildemberg; DIA) [19, 60, 61, 66] and 6 considered only membrane staining (HER2‐Score; Takei; Casarini; Chinezu; IRS; H‐score) [16, 17, 18, 20, 59, 62]. There is still debate regarding the interpretation of the specific location of somatostatin receptor staining in immunohistochemical studies. Reubi et al. identified subcellular localization (cytoplasmic staining) in NET, validated by mRNA expression and associated this subcellular location with the receptor internalization [75]. However, somatostatin ligands act mainly on receptors located in the cell membrane, which confers membrane staining, and which was considered the true predictor of response to drug treatment [75]. In this sense, in one study pure SST2 cytoplasm staining showed poor correlation with scintigraphy. It should be noted that in this large series there was only one single pituitary adenoma [60]. Iacovazzo et al. analyzed SST2 and SST5, also considering the subcellular location, and found that no somatotropinomas with exclusively cytoplasmic expression of SST2 responded to SRL [70].

Campana et al. evaluated both cytoplasmic and membrane positivity and raised the hypothesis that consideration of exclusively membrane labeling may underestimate the real number of receptors in tumor cells, especially in patients who received preoperative treatment with somatostatin ligands [66]. In fact, different groups reported a lower expression of SST2 observed in patients treated with SRL as compared to treatment naive patients, and which could be explained by the internalization of receptors induced by ligands [19, 67, 76]. It has been shown that internalized receptors are inactive and membrane localization is most accurately correlated with the activity of SST [62].

The most reproduced scoring system was the IRS [16, 63], which was applied by four independent groups, followed by the one used by Takei et al. [17, 69] and the Volante score [60, 70], reproduced each by one group. In our initial studies as mentioned above, our group used a scoring system considering only the percentage of positive cells and compared it to other methods of SST detection [61]. In accordance with other authors, we have tested and validated the IRS, which was considered by other authors as the best to predict the response to treatment with SRL [65]. Therefore, it has been the standard evaluation method we use in our routine.

**FIGURE 7:**
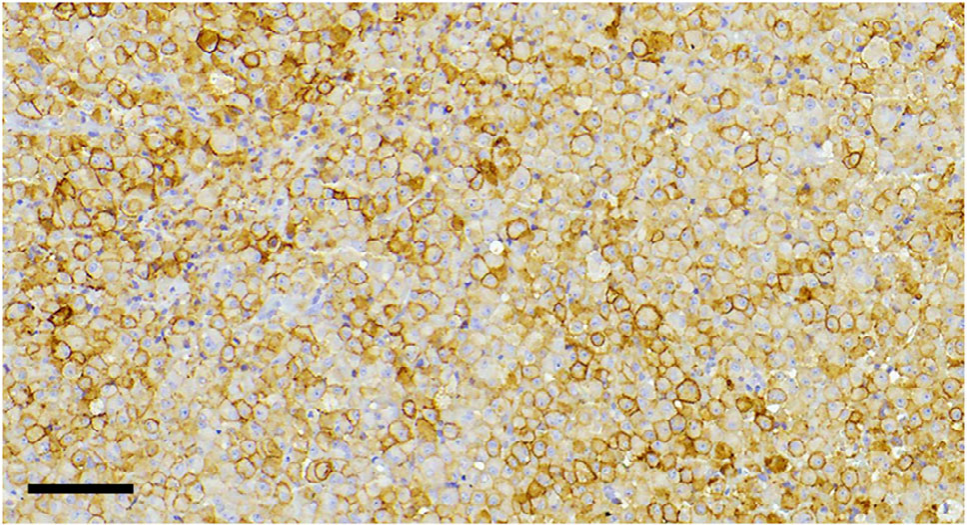
Heterogeneity of SST2 expression. SST2 IHC expression in a somatotroph adenoma, with variable intensity within the same tumor, resulting in a difficult scoring. Scale bar measures 90 μm.

Even though in most cases of somatotroph adenomas SST2 and SST5 immunostains are straightforward to interpret, as mentioned above, one pitfall we confront in our routine is the heterogeneity of the intensity of immunostaining in different areas of a same tumor as shown in Figure 7. Determining the IRS in samples with heterogeneous staining intensity can be problematic. Casar‐Borota et al. have tried to overcome this difficulty by considering the most prevalent intensity on the slide to assign a final IRS scoring [67]. The histoscore has the advantage of integrating proportionally all cells with respective staining intensity [18]. Moreover, the optimal cutoff for the IRS—equaling 5, as proposed by Gatto et al., or equaling 7, as indicated by Casar‐Borota and Ilie et al.—to be used as predictive of response to SRL is still an open question [16, 67, 68].

Most of the scoring systems have applied a semiquantitative analysis, subdividing in 3 or 4 scores, except for the H‐score and the DIA, that considered a continuous variable to quantify the immunohistochemical expression and which renders a potentially more accurate quantification of SSTs [18, 66].

Applying the DIA to evaluate SST2 expression has shown promising results [66]. However, this method has still a potential source of bias, as there are two manual steps before the actual DIA. Nonetheless, their results are in line with recent publications, which have shown that applying digital analysis and artificial intelligence assistance improves the accuracy of pathological analysis, particularly for scoring and quantifying markers [77, 78]. Further studies are necessary to validate these findings.

Few studies have directly compared different scoring systems. Kaemmerer et al. compared the HER 2‐score with the IRS and suggested that the IRS should be more adequate to evaluate the SST because of the heterogeneous cytoplasmatic staining expression, but there was no statistic difference between them [79]. Kasajima et al. had reported interobserver and interlaboratory agreement for HER 2‐score and the volante‐score by comparing these two evaluation methods in neuroendocrine tumors [80]. However, pituitary adenomas were not included in these two studies. Campana et al. showed an interobserver reproducibility and compared the DIA and the IRS methods and found a good correlation between them, showing nonetheless a higher accuracy with the DIA [66]. This is the single study which directly addressed the question of the reproducibility of the scoring system for SST2 in pituitary adenomas.

Concerning the assessment of SST5 in the literature the analyses have been essentially done using the scoring systems dedicated to SST2 [17, 18, 20, 59, 60, 62, 63, 64, 65, 67, 69, 70]—details are shown in Table S3.

**FIGURE 8:**
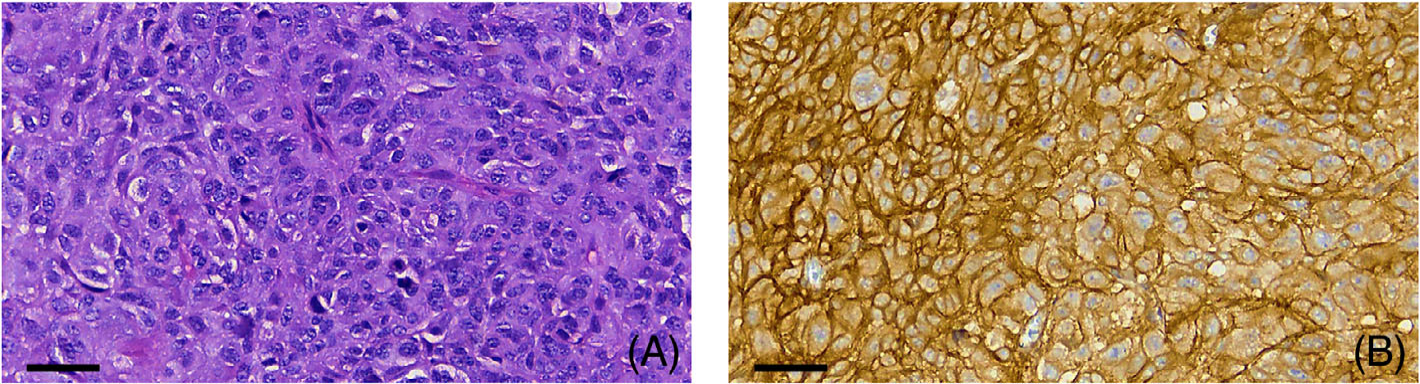
SST5 in a corticotroph adenoma. (A) Corticotroph tumor displaying basophilic appearance in hematoxylin & eosin‐stained section. (B) Strong and diffuse IHC expression of SST5. Scale bars measure 40 μm.

**FIGURE 9:**
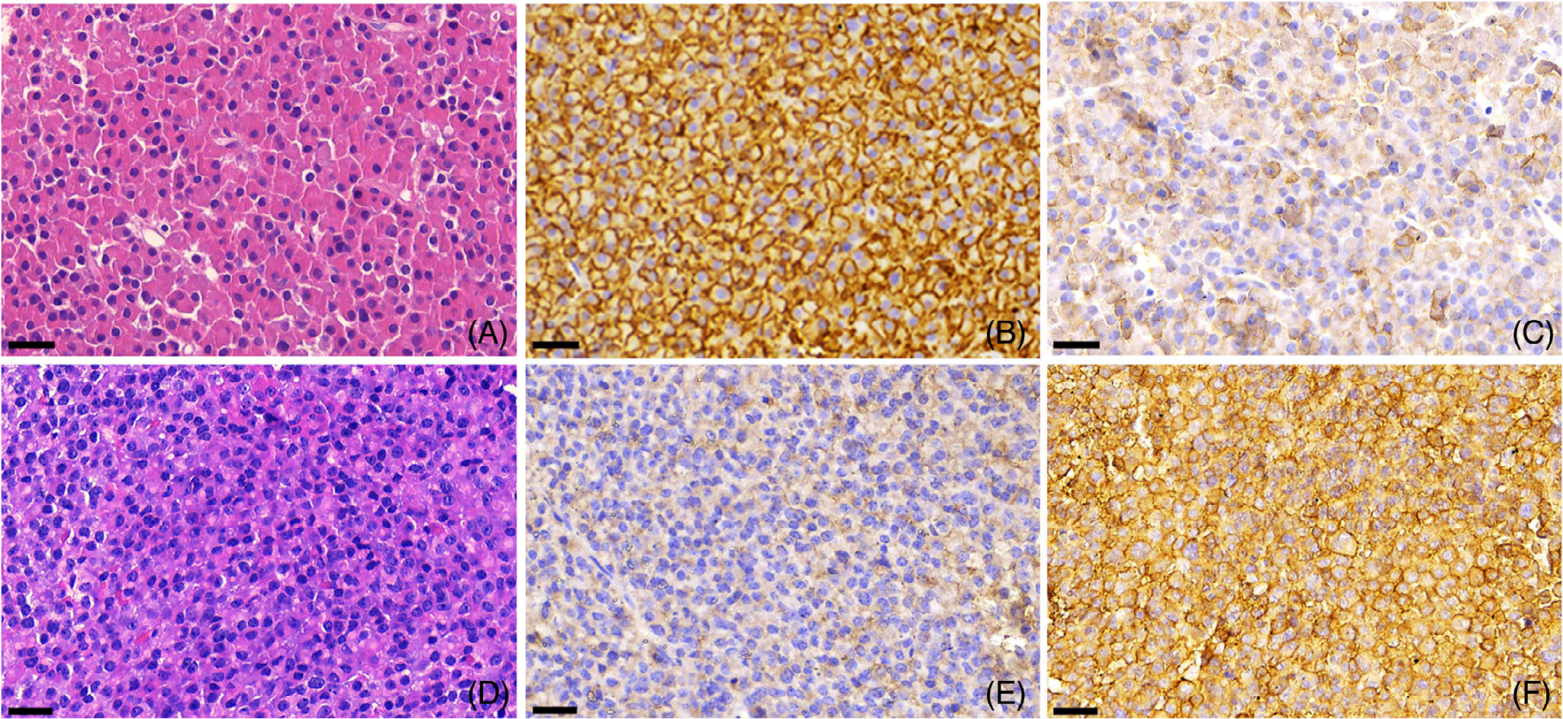
(A – C) A Densely Granulated (DG) somatotroph adenoma from a patient who responded to first generation SRL. A: Hematoxylin & eosin‐stained section showing tumor cells with densely eosinophilic cytoplasm. B: High SST2 IHC expression (IRS = 12). C: Lower SST5 IHC expression (IRS = 6). (D – F) A Sparsely Granulated (SG) somatotroph adenoma from a patient who did not respond to first generation SRL and responded to pasireotide. (D) Hematoxylin & eosin stained section showing tumor cells with chromophobe or lightly eosinophilic cytoplasm. (E) Low SST2 IHC expression (IRS = 4). (F) High SST5 IHC expression (IRS = 12). All scale bars measure 30 μm.

As corticotroph and sparsely granulated somatotroph adenomas tend to express SST5, which can indicate a better treatment response to pasireotide (Figures 8 and 9) [65, 81], specific studies validating and uniformizing the SST5 scoring systems would be of interest.

## 4 SST IN GH‐SECRETING PITUITARY ADENOMAS

Most somatotroph adenomas show expression of SST2 and SST5, for instance our group observed 75% and 56% significant positivity, respectively [65], a somewhat higher rate using the same criteria as Gatto et al., who reported 52% positivity for SST2 [16]. Densely granulated (DG) /intermediate adenomas tend to express more SST2 and are therefore generally more responsive to treatment with somatostatin receptor ligands (SRLs). Although in one study there was no difference in the expression of SST5 between sparsely and densely granulated somatotroph adenomas [62], other studies have reported that sparsely granulated (SG) adenomas usually express higher SST5 [63, 82] (Figure 9). Chinezu et al. showed that SST2 expression was lower in silent somatotroph tumors compared to tumors from patients with acromegaly, but there was no significant difference in SST5 expression between them [64].

Recently, DG somatotroph adenomas coexpressing the transcription factors PIT1 and SF1 have been shown to have a distinct molecular and epigenetic profile when compared with other somatotroph adenomas, and a meta‐analysis indicated that they respond less favorably to SRL than somatotroph tumors which do not express SF1, and both have a better prognosis than SG somatotroph adenomas [83]. Nevertheless, another group demonstrated that 7/8 somatotroph tumors coexpressing PIT1 and SF1 expressed strongly SST2, but weakly SST5 and two cases (one with residual tumor and one with mild biochemical persistence) treated with SRL achieved remission for several years [75].

The most recent classification of WHO for endocrine tumors (2022) includes seven different subtypes of PIT1 lineage tumors which can variably express GH and can also be associated with acromegaly, namely, DG and SG somatotroph, mixed lactotroph‐somatotroph, mammosomatotroph, acidophil stem cell, mature plurihormonal PIT1‐lineage, and immature PIT1‐lineage adenomas [11]. Apart from SG and DG somatotroph adenomas, in the literature there are no studies exploring the particular features of expression pattern of SSTs in each of these different types of tumors.

## 5 CLINICAL SIGNIFICANCE OF QUANTIFICATION OF SOMATOSTATIN RECEPTORS IN PITUITARY ADENOMAS AND FUTURE PERSPECTIVES

In acromegaly, fg‐SRL are associated with biochemical control (normalization of insulin like growth factor type I—IGF‐I) in approximately 50% of patients and induce significant tumor shrinkage (>20%) in over 60% of patients [15].

Several factors have been evaluated as biomarkers of response to these drugs, which include characteristics from the patient, tumor, such as sex, age, tumor invasiveness and MRI signal, tumor granulation pattern, expression of Ki‐67, zinc‐finger protein which regulates apoptosis and cell‐cycle arrest 1 (ZAC‐1), E‐cadherin and aryl hydrocarbon receptor‐interacting protein (AIP), and SST [15, 74, 84]. Among them all, SST2 expression in somatotropinomas is recognized as the most well‐established biomarker of response to fg‐SRL [15].

Gatto et al. found that patients who achieved IGF‐I normalization with treatment with fg‐SRL showed higher SST2 expression than those who did not. Also, a high SST2 expression (IRS > 5) had a sensitivity of 86% and a specificity of 91% in predicting IGF‐I normalization [16]. Our group found 94% sensitivity, but only 35% specificity [65]. Other studies evaluated SST expression and correlated it with response to fg‐SRL, almost invariably demonstrating a positive correlation [18, 63, 67, 68, 69, 70].

Pasireotide normalized IGF‐I levels in nearly 40% in patients not previously medically treated, and 80% presented tumor shrinkage [85]. In patients resistant to fg‐SRL, it induced biochemical remission (mean GH < 1.0 ng/mL and IGF‐I normalization) in 37% of patients after an average of 5.8 years [86]. In an even longer follow‐up (up to 11.4 years of treatment), our group showed in a series of 50 patients, most of them resistant to fg‐SRL, that pasireotide was able to normalize IGF‐I levels in 54% of patients and induce tumor reduction (>25%) in 63% [87].

Data in respect to biomarkers of response to pasireotide are scarcer. Iacovazzo et al. found that, in patients with acromegaly resistant to fg‐SRL, those presenting tumors with low to absent SST5 expression showed no response to pasireotide. On the other hand, among those with moderate to high SST5 expression, five out of seven were responsive [70]. In another study, including patients pretreated or not with fg‐SRL, pasireotide responsiveness was correlated with SST2 expression [63]. In fact, preclinical data indicate that in GH secreting adenomas, pasireotide effects seems to be mediated mainly by SST2 [88].

SST2 expression presents a high correlation with response to fg‐SRL; however, its accuracy is not optimal. In our data, we found an accuracy of 55%. So, combinations of biomarkers have been used to try to increase the accuracy of response prediction. Our group, in a large multicenter study, combined seven biomarkers using machine learning techniques to develop a model to predict response to fg‐SRL. These biomarkers were SST2 and SST5, granulation pattern, sex, age, and baseline GH and IGF‐I levels. The model was able to predict response with a 71.4% sensitivity, 93.3% specificity, 83.3% positive predictive value, and 87.5% negative predictive value. The overall accuracy was 86.3%, which was much higher than the accuracy of SST2 expression alone (55%). Other models including SST2 expression and additional biomarkers have also been described [89, 90].

Finally, a multicenter study evaluated prospectively 49 patients with acromegaly medically treated with fg‐SRL followed by pasireotide after non‐curative pituitary surgery [68]. The authors demonstrated that IGF‐I decrease after treatment with fg‐SRL was positively correlated with SST2 protein expression evaluated with IRS. Also, controlled patients more frequently presented densely granulated tumors.

Currently, in acromegaly, first line therapy is transsphenoidal surgery for the majority of patients [91]. For those who are not cured by surgery, or for the selected ones for whom surgery was not indicated, fg‐SRL are the cornerstone of treatment [15, 91]. Other treatments, such as dopamine agonists, growth hormone receptor antagonist, pasireotide, and radiotherapy are usually indicated in those not controlled with fg‐SRL [91]. However, because of the high costs of most of the drugs used to treat acromegaly, they may not be widely available, and radiotherapy may be used in such cases.

To date, medical management of secreting pituitary adenomas, in particular acromegaly, has been based on a trial‐and‐error approach. Efforts are being made to advance toward a personalized approach, based on biomarkers, among which SST expression is the most consistent [15, 65, 92, 93]. This approach would lead to a more efficient treatment, avoiding the use of drugs which are not efficient to a particular patient. Considering that the medical management of acromegaly is lifelong and based on high‐cost drugs, a personalized approach would contribute to decrease cost burden on health systems. However, the best combination of biomarkers, and how they should be applied is still a matter of research.

In a nutshell, there are substantial data in the literature supporting the evaluation of the immunohistochemical evaluation of SST2 as a predictor of response to SRL. Nonetheless, as previously discussed, although the IRS has been the most consistently used system, and has proven to be a valuable tool, a universal scoring system is still lacking [94, 95]. Interesting results with digital analyses of SST2 IHC have been recently reported, but not yet validated [66]. An international multicenter study applying homogeneous immunostaining protocols and one same reproducible scoring system, ideally with the aid of digital pathology and artificial intelligence tools, would be a desirable scenario for pathologists to validate a precise and consistent system to evaluate SST2 expression, and help clinicians to define the optimal therapeutic strategy for the patients with somatotroph adenomas.

Note: Immunostains for SST2 and SST5 shown in Figures 1, 6, 7, 8, 9 were performed using UMB‐1 and UMB‐4 antibodies (UMB‐1, 1:5000, Abcam, Cambridge, UK, cat. number ab 134152; UMB‐4, 1:2000, Abcam, cat. number ab 109495) as previously described [96].

## AUTHOR CONTRIBUTIONS

FA and MRG conceived the manuscript. LFB, RSD and FA were responsible for figure generation.All authors actively participated in the writing and editing of the manuscript.

## CONFLICT OF INTEREST STATEMENT

MRG has received speaker fees from Recordati Rare Diseases, Ipsen, Crinetics Pharmaceuticals, and Novo Nordisk and attended advisory boards for Novo Nordisk, Recordati Rare Diseases, and Crinetics Pharmaceuticals. LEW has received speaker fees from Novartis and Ipsen and participated in advisory boards for Crinetics Pharmaceuticals.

## Supporting information


Table S1.

Table S2.

Table S3.

## Data Availability

The data that support the findings of this study are available from the corresponding author upon reasonable request.
